# Human saliva, plasma and breast milk exosomes contain RNA: uptake by macrophages

**DOI:** 10.1186/1479-5876-9-9

**Published:** 2011-01-14

**Authors:** Cecilia Lässer, Vesta Seyed Alikhani, Karin Ekström, Maria Eldh, Patricia Torregrosa Paredes, Apostolos Bossios, Margareta Sjöstrand, Susanne Gabrielsson, Jan Lötvall, Hadi Valadi

**Affiliations:** 1 Krefting Research Centre, Sahlgrenska Academy, University of Gothenburg, Box 424, 405 30 Gothenburg, Sweden; 2 Department of Medicine, Clinical Allergy Research Unit, Karolinska University Hospital Solna, Stockholm, Sweden; 3 Dept. of Rheumatology and Inflammation Research, Sahlgrenska Academy, University of Gothenburg, Guldhedsgatan 10A, 413 46 Gothenburg, Sweden

## Abstract

**Background:**

Exosomes are 30-100 nm membrane vesicles of endocytic origin produced by numerous cells. They can mediate diverse biological functions, including antigen presentation. Exosomes have recently been shown to contain functional RNA, which can be delivered to other cells. Exosomes may thus mediate biological functions either by surface-to-surface interactions with cells, or by the delivery of functional RNA to cells. Our aim was therefore to determine the presence of RNA in exosomes from human saliva, plasma and breast milk and whether these exosomes can be taken up by macrophages.

**Method:**

Exosomes were purified from human saliva, plasma and breast milk using ultracentrifugation and filtration steps. Exosomes were detected by electron microscopy and examined by flow cytometry. Flow cytometry was performed by capturing the exosomes on anti-MHC class II coated beads, and further stain with anti-CD9, anti-CD63 or anti-CD81. Breast milk exosomes were further analysed for the presence of Hsc70, CD81 and calnexin by Western blot. Total RNA was detected with a Bioanalyzer and mRNA was identified by the synthesis of cDNA using an oligo (dT) primer and analysed with a Bioanalyzer. The uptake of PKH67-labelled saliva and breast milk exosomes by macrophages was examined by measuring fluorescence using flow cytometry and fluorescence microscopy.

**Results:**

RNA was detected in exosomes from all three body fluids. A portion of the detected RNA in plasma exosomes was characterised as mRNA. Our result extends the characterisation of exosomes in healthy humans and confirms the presence of RNA in human saliva and plasma exosomes and reports for the first time the presence of RNA in breast milk exosomes. Our results also show that the saliva and breast milk exosomes can be taken up by human macrophages.

**Conclusions:**

Exosomes in saliva, plasma and breast milk all contain RNA, confirming previous findings that exosomes from several sources contain RNA. Furthermore, exosomes are readily taken up by macrophages, supporting the notion that exosomal RNA can be shuttled between cells.

## Background

Exosomes are small membrane vesicles (30-100 nm) of endocytic origin that are released from the producing cell into the extracellular environment [1]. Many cells in the body have the capacity to produce and release exosomes to their surrounding environment, including dendritic cells, B cells, T cells, mast cells, tumour cells and epithelial cells [2-7]. Exosomes are also present in body fluids including plasma, urine, saliva, malignant effusions, synovial fluid, breast milk, bronchoalveolar lavage fluid and epididymal fluid [8-15] indicating importance *in vivo*. Until now, exosomes have been implicated primarily in antigen presentation, as they often express several proteins involved in cell adhesion and co-stimulation including ICAM-1, CD86, CD63 and CD82, MHC class I and MHC class II [1]. These immunological functions have led to the development of anti-tumour vaccines based on exosomes, which are currently in early clinical development [16,17].

Exosomes have been proposed to signal by both the binding to cell surface receptors through adhesion molecules [3] and by fusion with or internalisation by the recipient cell, potentially donating their own cytoplasm to the recipient cell [18,19]. The latter implies that exosomes may have mechanisms that are different to their function in the immune system. We have recently discovered substantial amounts of RNA in exosomes derived from mast cells [20], which have the capacity to donate their RNA to other cells and can subsequently affect the protein production of a recipient cell. This argues that RNA can be transferred between mammalian cells by an extracellular exosome based transport mechanism, which has vast implications in the understanding of cell communication, regulation and signalling, in addition to extensive therapeutic potential in many diseases. Therefore, studies to determine the presence of RNA in exosomes harvested from humans *in vivo *are of high priority.

As human plasma, saliva and breast milk all contain exosomes [8,12,15], the aims of the current study were to determine whether these exosomes contain RNA and whether they can be taken up by other cells, which would support the concept that shuttling of RNA may occur in humans.

## Methods

### Exosome purification from saliva

Saliva from healthy humans was collected in Falcon tubes on ice, during a period of no eating or drinking and pooled together. For the RNA isolation experiment, 100 μl of the protease inhibitor Complete Mini (Roche Diagnostics Scandinavia AB, Bromma, Sweden) and 800 units of RNase inhibitor RiboLock Ribonuclease Inhibitor (Fermentas, St. Leon-Rot, Germany) were added per 20 ml of saliva. For the flow cytometry, electron microscopy and uptake experiments no inhibitors were added to the tubes. The saliva was diluted 1:1 with phosphate buffered saline (PBS) and centrifuged at 16 500 × g for 20 min to remove cells and debris. The supernatant was filtered through a 0.2 μm VWR^® ^Vacuum Filtration System (VWR International, West Chester, PA, USA), before ultracentrifugation (Ti70 or Ti45 rotor, Beckman Coulter, Brea, CA, USA) at 120 000 × g for 70 min to pellet the exosomes.

### Exosome purification from blood plasma

A volume of 450-500 ml of blood was collected from donors. Plasma was derived from heparinised blood by centrifugation at 1 800 × g for 10 min. Further centrifugation at 29 500 × g for 20 min was performed to pellet any remaining cells and debris. The supernatant was then filtered through a 0.2 μm VWR^® ^Vacuum Filtration System, followed by ultracentrifugation at 120 000 × g for 90 min to pellet the exosomes.

### Exosome purification from breast milk

Human breast milk was collected from healthy mothers, immediately stored at -20ºC and later transferred to the laboratory and stored at -80 ºC. To remove cells and debris, the breast milk was first centrifuged at 300 × g for 10 min, followed by centrifugation at 16 500 × g for 20 min. The supernatant was then filtered through a 0.2 μm VWR^® ^Vacuum Filtration System, followed by ultracentrifugation at 120 000 × g for 70 min to pellet the exosomes.

### Electron microscopy

Exosomes from saliva, plasma and breast milk were isolated as described above, washed in PBS to further purify the sample, filtered, and ultracentrifuged again at 120 000 × g for 70 min to re-pellet the exosomes. The exosome pellet was resuspended in PBS and loaded onto formvar carbon coated grids (Ted Pella Inc, Redding, USA). Next, the exosomes were fixed in 2% paraformaldehyde and washed. The exosomes were immunostained with anti-CD63 antibody (BD Bioscience, Erembodegem, Belgium) or isotype control (Sigma-Aldrich, St Louis, MO, USA), followed by staining with a 10 nm gold-labelled secondary antibody (Sigma-Aldrich). The exosomes were subsequently fixed in 2.5% glutaraldehyde, washed, contrasted in 2% uranyl acetate and embedded in a mixture of uranyl acetate (0.8%) and methyl cellulose (0.13%). The preparations were examined in a LEO 912AB Omega electron microscope (Carl Zeiss NTS, Jena, Germany).

### Flow cytometry of exosomes

Isolated saliva, plasma or breast milk exosomes were resuspended in PBS and loaded onto anti-MHC class II coated beads (custom-made by Dynal, part of Invitrogen Ltd, Paisley, UK). The anti-MHC class II coated beads (8 × 10^4^) were mixed with a minimum of 50 μg of exosomal protein, before being incubated overnight at 4ºC with gentle agitation. The bead-exosome complexes were washed twice in PBS with 3% Fetal Bovine Serum (FBS). Prior to use, the FBS was ultracentrifuged at 120 000 × g for 1.5 hours, to eliminate serum exosomes. The bead-exosome complexes were resuspended in IgG (Sigma-Aldrich) and incubated for 15 min at room temperature, before being washed twice more, as above. The tetraspanins CD9, CD63 and CD81, known to be enriched in exosomes, were used as markers for exosomes. The bead-exosome complexes were incubated with PE-labelled anti-CD9 (clone M-L13), anti-CD63 (clone H5C6), anti-CD81 (clone JS-81) or the corresponding isotype control (all antibodies were from BD Biosciences) for 40 min at room temperature with agitation and washed three times before analysis. As a control for unspecific binding of the antibodies to the beads, beads were stained with all three antibodies without the addition of exosomes and showed no difference when compared to exosome coated beads stained with the isotype control. The samples were then acquired in a FACScan or FACSAria (BD Biosciences) and analysed using the FlowJo Software (Tri Star Inc, Ashland, OR, USA).

### Western blot analysis of breast milk exosomal proteins

Isolated breast milk exosomes were re-suspended in PBS and ultracentrifuged at 120 000 × g for 70 min to be re-pelleted before dissolved in ProteoJET Mammalian Cell Lysis Reagent (Fermentas). For extraction of total protein, the sample was incubated at room temperature for 10 min on a shaker, sonicated for 5 min and vortexed, before being centrifuged at 13 000 × g for 10 min. The protein content of the supernatant was measured with a spectrophotometer at 750 nm utilising the D_c _Protein Assay reagent A and B (Bio-Rad Laboratories, Hercules, CA, USA). 100 μg proteins from the supernatant were loaded per well onto a 10% acrylamide gel. Monocyte derived macrophages from buffy coat were used as a control. The proteins were blotted onto a nitrocellulose membrane (Bio-Rad Laboratories) overnight at 4°C. The membrane was blocked with 0.5% Blotting Grade Blocker Non-Fat Dry Milk (Bio-Rad Laboratories) in TBS for 2 h, before washed 3 × 5 min in TBS-Tween (used for all the washes throughout the Western blot experiment). The membrane was then incubated with either anti-calnexin (1:1000) (Santa Cruz Biotechnology, Santa Cruz, CA, USA), anti-Hsc70 (1:1000) (Enzo Life Science, Farmingdale, NY, USA) or anti-CD81 (1:800) (Santa Cruz) diluted in 0.25% non-fat dry milk in TBS-Tween for 2 h. The membrane was washed 3 × 5 min before incubated with the secondary antibody for 2 h. The secondary antibodies used were goat F(ab)_2 _anti-rabbit IgG (HRP conjugated) for the calnexin and CD81 (1:5000) (Harlan Sera-Lab, Loughborough, UK) and rabbit F(ab)_2 _anti-Rat IgG (HRP conjugated) for the Hsc70 (1:4000) (Southern Biotech, Birmingham, AL, USA) diluted in 0.25% non-fat dry milk powder in TBS-Tween. The membrane was washed 3 × 5 min, before being analysed with the Amersham™ ECL Plus™ Western Blotting Detection System (GE Healthcare, Uppsala, Sweden) and a VersaDoc 4000 MP (Bio-Rad Laboratories).

### RNA isolation and detection

RNA was isolated using Trizol^® ^(Invitrogen) according to the manufacturer's protocol and dissolved in DEPC H_2_O (Fermentas). For detection of RNA, an Agilent 2100 Bioanalyzer (Agilent Technologies Sweden AB, Kista, Sweden) was utilised for all samples. The exosomal RNA was compared with cellular RNA from the human mast cell line HMC-1. The HMC-1 cells (Dr J. Butterfield, Mayo Clinic, Rochester, MN, USA) were cultured in a 37ºC humidified incubator with 5% CO_2_, in complete medium consisting of Iscove's Modified Dulbecco's Medium (IMDM) supplemented with 10% FBS, 100 units/ml penicillin, 100 μg/ml streptomycin, 2 mM L-glutamine and 1.2 mM/ml alfa-thioglycerol (all reagents from Sigma-Aldrich).

For the detection of mRNA in exosomes, the total RNA isolated was converted to cDNA using RevertAid™ H Minus First Strand cDNA Synthesis Kit (Fermentas) and the oligo (dT) primer. The second strand of the cDNA was synthesised by adding 10 μl of 10 × DNA polymerase 1 reaction buffer, 4 μl of DNA polymerase 1, 5 μl of T4 DNA ligase and 61 μl of DEPC water (all reagents were from Fermentas) to the first strand of cDNA product. The sample was incubated at 14ºC for 2 h before the reaction was stopped by incubation at 70ºC for 10 min. The detection of cDNA was performed using a Bioanalyzer.

### Exosome staining

Saliva and breast milk exosomes were isolated as described above, and further purified by being dissolved in PBS and ultracentrifuged at 120 000 × g for 70 min. The exosomes were labelled with PKH67 Green Fluorescent Cell Linker Kit for General Cell Membrane Labelling (Sigma-Aldrich) according to the manufacturer's protocol, with minor modifications in the washing process. Briefly, the exosomes were diluted in PBS before 1 ml of Diluent C was added. As a control, 1 ml of Diluent C with the same volume of PBS was used. 4 μl of PKH67 dye was added to 1 ml of Diluent C before being added to the exosomes and the control. The samples were mixed gently for 4 min before 2 ml of 1% BSA was added to bind the excess dye. The samples were then transferred to 300 kDa Vivaspin filters (Sartorius Stedim Biotech GmbH, Goettingen, Germany) and centrifuged at 4000 × g. The sample were washed 3 times with 5 ml of PBS before being transferred to new 300 kDa Vivaspin filters and washed twice with 5 ml IMDM (Sigma-Aldrich).

### Uptake of saliva and breast milk exosomes by macrophages

Peripheral mononuclear cells (PBMCs) were isolated from buffy coat using Leucosep^® ^Tubes (Greiner Bio-One GmbH, Frickenhausen, Germany), according to the manufacturer's protocol. The PBMCs were washed repeatedly with 2 mM EDTA in PBS, before being dissolved in 0.5% BSA and 2 mM EDTA in PBS. Monocytes were isolated from PBMCs using a Monocyte Isolation Kit II (Miltenyi Biotec Gmbh, Bergisch Glagbach, Germany) according to the manufacturer's protocol. The purity of the monocytes was determined with a FACSAria by the detection of CD14 (clone MΦP9, BD Biosciences). To allow for differentiation into macrophages, the monocytes were cultured for 7 days in a 37ºC humidified incubator with 5% CO_2_, in complete medium consisting of IMDM supplemented with 10% FBS, 100 units/ml penicillin, 100 μg/ml streptomycin, 2 mM L-glutamine, 110 μg/ml sodium pyruvate (all reagents were from Sigma-Aldrich) and 10 ng/ml GM-CSF (R&D Systems, Minneapolis, MN, USA). The FBS was ultracentrifuged prior to use to eliminate serum exosomes. For analysis with flow cytometry cells were cultured in 96-well plates and for fluorescence microscopy, the cells were cultured in 8-well Permanox Slides (Thermo Fisher Scientific, New York, USA).

10 μg of the PKH67 labelled exosomes or the same volume of the PKH67-PBS control was added per 200 000 macrophages and incubated for 2 h at either 37ºC or 4ºC. The binding of the exosomes to the macrophages was analysed with a FACSAria and visualised with fluorescence microscope (Zeiss Axioplan 2, Carl Zeiss, Jena, Germany). For analysis with flow cytometry the cells were washed twice with PBS, treated with a 0.25% trypsin-EDTA solution (Sigma-Aldrich) and washed twice with 1% FBS in PBS before acquired in FACSAria and analysed with FlowJo software. For fluorescence microscopy, the cells were washed twice with PBS, fixed with 4% formaldehyde for 15 min and washed twice with PBS before being mounted with Vectashield (Vector Laboratories Inc., Burlingame, USA) with 3% 7-ADD (BD Biosciences) to label nuclei.

## Results

### Human saliva, plasma and breast milk contain exosomes

**Figure 1:**
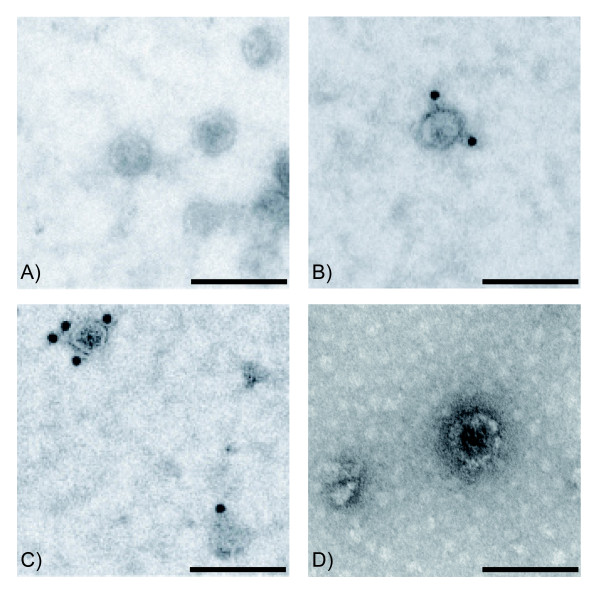
**Exosomes from saliva, plasma and breast milk detected with electron microscopy. **Exosomes from human saliva (A, B), plasma (C) and breast milk (D) were examined in the electron microscope. No isotype control antibody (A), but anti-CD63 antibody (B-D), was detected by 10 nm gold labelled secondary antibody. The scale bars represent 100 nm.

**Figure 2:**
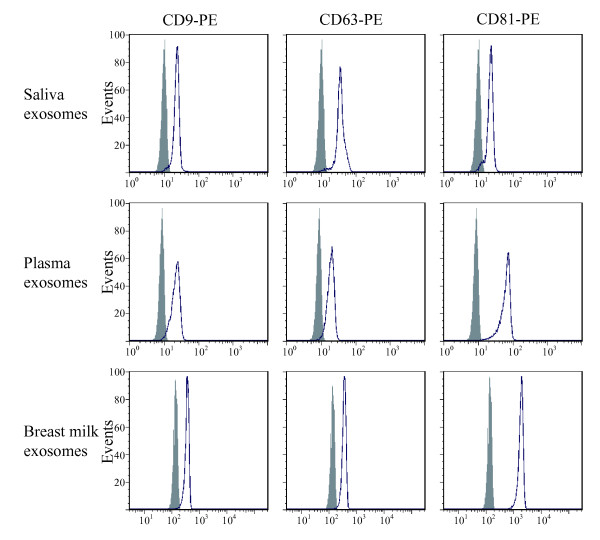
**Flow cytometry detection of surface molecules on exosomes from saliva, plasma and breast milk. **Exosomes from saliva, plasma and breast milk captured on anti-MHC class II beads were immunostained by using monoclonal antibodies against the tetraspanins CD9, CD63 and CD81 and analysed by flow cytometry. The antibodies (open peaks) were compared with their appropriate isotype controls (filled peaks).

**Figure 3:**
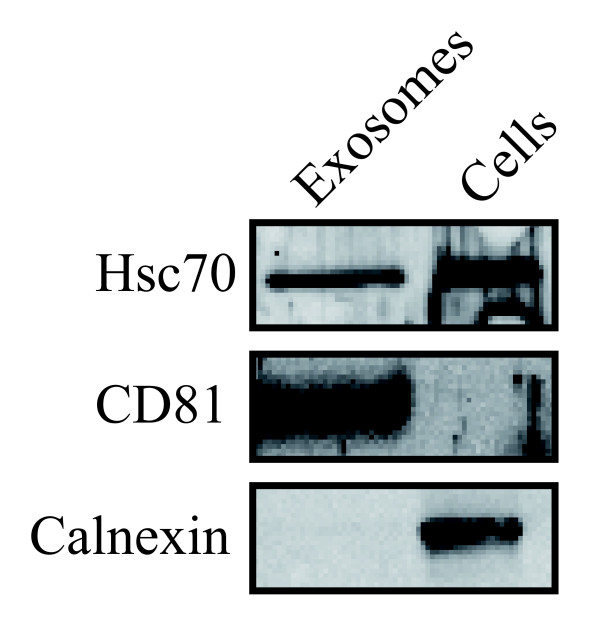
**Characterisation of breast milk exosomes by Western blot. **The exosomal proteins from breast milk exosomes were loaded onto a 10% acrylamide gel and transferred to a nitrocellulose membrane. The breast milk exosomes are positive for Hsc70 and CD81, but negative for the endoplasmic reticulum protein, calnexin. Macrophage protein ("Cells") was used as positive control.

Exosomes from saliva, plasma and breast milk were identified using electron microscopy (Figure 1A-D) and exosomes from all sources were positive for CD63, using immunogold staining (Figure 1B-D). Furthermore, flow cytometry of saliva, plasma and breast milk exosomes captured on anti-MHC class II coated beads revealed the presence of CD9, CD63 and CD81 on exosomes from all three sources (Figure 2). Breast milk exosomes were further characterised by Western blotting and was shown to be positive for Hsc70 and CD81, but negative for the endoplasmic reticulum marker calnexin (Figure 3).

### Human exosomes contain RNA

**Figure 4:**
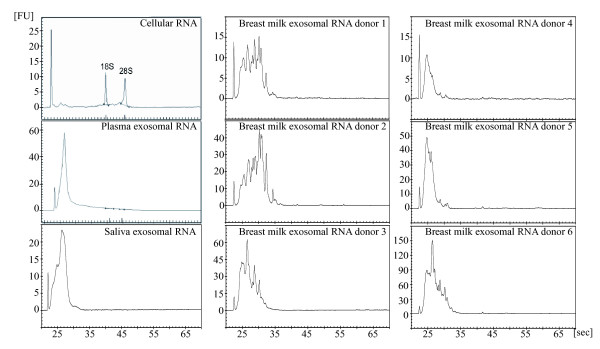
**Exosomal RNA analysed using a Bioanalyzer. **Total RNA was isolated from saliva, plasma and breast milk exosomes using Trizol^® ^and analysed with a Bioanalyzer. The results show that exosomes from human saliva, plasma and breast milk contain a dissimilar RNA content compared to cellular RNA from HMC-1 cells, as exosomes contain little or no ribosomal RNA.

The RNA content of the saliva, plasma and breast milk exosomes was analysed using a Bioanalyzer instrument, which revealed that all three types of exosomes contain RNA, with little or no ribosomal RNA (18S- and 28S- rRNA) (Figure 4). The pattern of exosomal RNA visualised in the Bioanalyzer differed substantially from HMC-1 cell RNA, which contain substantial amounts of ribosomal RNA (Figure 4).

**Figure 5:**
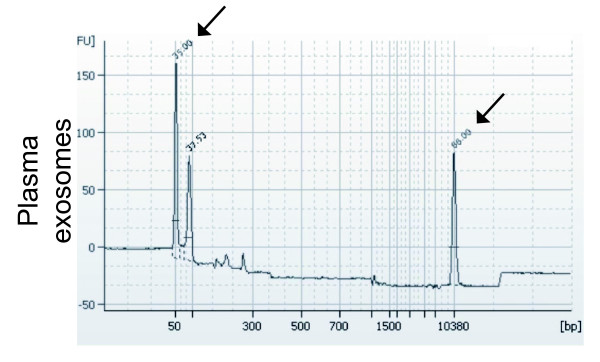
**Detection of mRNA in plasma exosomes using a Bioanalyzer. **The exosomal RNA was transcribed to cDNA using an oligo (dT) primer. The results show that a portion of the RNA in plasma exosomes is mRNA. Arrows show the peaks for the lower and upper markers. The peaks in between these markers indicate the presence of cDNA synthesised from plasma exosomal RNA.

We also confirmed the presence of polyadenylated RNA in exosomes from plasma, by synthesising cDNA using an oligo (dT) primer (Figure 5). However, cDNA could not be synthesised from exosomal RNA extracted from saliva or breast milk, using the same method (data not shown).

### Human macrophages take up human saliva and breast milk exosomes

**Figure 6:**
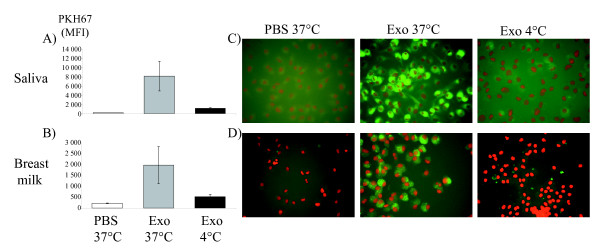
**Uptake of saliva and breast milk exosomes by human macrophages. **10 μg of the PKH67-labelled saliva exosomes, PKH67-labelled breast milk exosomes or a PKH67-PBS control were added per 200 000 macrophages and incubated at 37ºC or 4ºC for 2 h. The uptake of the fluorescently labelled saliva and breast milk exosomes by macrophages was detected with both flow cytometry (A and B respectively) and fluorescence microscopy (C and D respectively). The uptake was reduced at 4ºC, indicating a biologically active uptake. In the fluorescence microscopy pictures (C and D), 7-AAD was used to detect the nucleus of the macrophages (red) and PKH67 was used to label the exosomes (green). MFI data are shown as mean ± SEM for saliva exosomes n = 3 and for breast milk exosomes n = 4.

To examine whether exosomes from human body fluids can be taken up by recipient cells, human saliva and breast milk exosomes were labelled with PKH67 dye (green) and added to cultures of human macrophages, derived from buffy coat monocytes (purity >94%). Flow cytometry showed an uptake of the exosomes by macrophages, shown by an increase of mean fluorescence intensity (MFI) for PKH67, compared with macrophages cultured with the PBS control, or cultured with exosomes at 4˚C (Figure 6A-B). The uptake of the fluorescent exosomes by the macrophages was also visualised using fluorescence microscopy (Figure 6C-D).

## Discussion

This study confirms the presence of exosomes in human saliva, plasma and breast milk, shown by both electron microscopy and flow cytometry. We demonstrate that exosomes from all three biological sources contain significant amounts of primarily short RNA, of which a portion is identified as mRNA in plasma exosomes. The study also shows uptake of saliva and breast milk exosomes by macrophages.

The vesicles isolated from saliva, plasma and breast milk, were shown by electron microscopy to have a diameter of 50-80 nm, which is comparable with previously identified exosomes [2-4]. Furthermore, immunogold staining showed that the exosomes were positive for the tetraspanin CD63, a commonly used exosome marker. Flow cytometry analysis further indirectly showed the presence of MHC class II on saliva, plasma and breast milk derived vesicles, as well as the presence of CD9, CD63 and CD81. While we acknowledge that viruses below 200 nm may constitute a small fraction of the exosome preparation, the EM analysis and detection of multiple exosomal proteins strongly suggests that the vesicles identified are exosomes and not other nano particles.

The current study confirms our original finding, that exosomes contain RNA [20] by clarifying that exosomes in different body fluids from healthy individuals also contain RNA. It was recently reported that exosomes from human plasma and saliva contain RNA [21-23], which further supports this conclusion. This study reports, for the first time, the presence of RNA in human breast milk exosomes, which implies that exosomes could deliver RNA from cells of the mother, to cells in the offspring.

Many compartments of the cell, besides the multivesicular bodies, can release vesicles. As the finding of RNA-containing exosomes in breast milk is novel, we confirmed that these were truly exosomes by showing the presence of Hsc70 and CD81, and the absence of the endoplasmatic reticulum protein, calnexin. As no calnexin was detected, this indicates that there is little, or no, contamination by endoplasmic reticulum-derived vesicles in the breast milk derived exosomes. Furthermore, breast milk exosomes has previously been shown to contain Hsc70 and CD81 [12], the detection of these molecules by Western blot on the breast milk derived exosomes isolated in this study served to further confirm their identification as exosomes. We therefore conclude that the RNA-containing vesicles found in breast milk are exosomes. We also confirmed our finding by detecting RNA-containing exosomes in breast milk from six different donors.

Exosomes from saliva and breast milk can be taken up by human macrophages, as shown by the uptake of fluorescently stained exosomes. It has been shown that other cells can take up exosomes in a similar way to macrophages [24,25], which indicates that this is a common feature of exosomes. The active uptake of the body fluid derived exosomes by recipient cells indicates *in vivo *relevance of exosome transfer. It has recently been shown that acidic conditions increases the uptake of tumour exosomes [19]. This could be important, as saliva exosomes may be taken up by cells in the acidic environment of the gastrointestinal tract.

The presences of RNA in exosomes from the three different human body fluids investigated, raises speculation about its importance in human biology. As exosomes can shuttle RNA between cells, it is not unreasonable to suggest that exosomes in plasma may be a vector for genetic communication between cells in different organs and that exosomes in breast milk may be an important vector for communication between mother and child via breastfeeding. We have previously found that the mRNA delivered from one mast cell to another mast cell via exosomal shuttle is functional [20]. However, it is possible that exosomal microRNA may have an extended capacity to affect a recipient cell by RNA interference [26]. It has also been shown in several studies of cancer patients, that plasma exosomes and/or similar vesicles, contain RNA [21,27,28]. Putatively, the RNA content in exosomes could be utilised as biological markers in different diseases. However, to reach that goal, extensive characterisation of the exosomal RNA from different diseases would be required, as well as in healthy humans.

In exosomes from plasma, we could detect the presence of mRNA, confirming our previous study showing presence of mRNA in mast cell exosomes [20], as well as confirming the studies showing the presence of mRNA in exosomes from human samples such as saliva and plasma [23,28]. Despite using the same method, the current study was unable to identify mRNA in the human saliva and breast milk exosomes. Importantly, the yield of RNA isolated from exosomes varies substantially, which strongly emphasises the need to optimise and standardise exosomal RNA isolation, which would then allow comparison between different exosome studies.

The biological significance of the shuttle of RNA between cells by exosomes has been previously determined in our original study [20], which showed that human mast cells can take up mouse mast cell exosomes and subsequently produce mouse proteins from the mRNA delivered in the exosomes. It is unclear whether biologically important shuttling of RNA is actually occurring in the human body, but our current study indirectly suggests that the potential for such a mechanism exists. It is likely that the most extensive shuttling of RNA would be occurring in the microenvironment around the cells producing and releasing the RNA-containing exosomes. However, the finding of RNA-containing exosomes in plasma implies that these at least theoretically could deliver RNA to distant cells.

Our novel discovery of RNA-containing exosomes in breast milk, suggests that these exosomes may transfer genetic signals from mother to child during breastfeeding. This increases both the complexity of the mother-to-child interaction and the complexity by which exosomes can function. Breast milk provides many health advantages to the child [29], but it has not yet been determined whether any such effect could be attributed to the exosome content in the breast milk. One effect of breast milk exosomes observed *in vitro *is the induction of T-regulatory (FOXP3 positive) cells [12], which leads to the speculation that exosomes could help the child develop immunological tolerance.

We cannot ignore the possibility that only a sub-population of saliva, plasma and breast milk exosomes contain RNA and extensive investigations will be required to determine exactly which cells produce exosomes containing functional RNA. The cellular sources of the exosomes in human plasma and breast milk are not clear, but the isolated exosomes are most likely released by a mixture of the immune competent cells present in the fluid and epithelial cells [2,3,7]. The origin of saliva exosomes has also not been determined, but it has been shown that primary cultures of salivary glands can release exosomes [30] which suggests that exosomes in saliva are at least partly derived from salivary gland epithelial cells.

## Conclusions

We have confirmed the presence of RNA in human plasma, saliva and breast milk exosomes, and have documented that exosomes from human saliva and breast milk can be taken up by human cells. As exosomes can deliver their RNA to the recipient cells, we suggest that human exosomes can deliver functional genetic signals to other cells. The finding of RNA-containing exosomes in saliva and breast milk, suggests that the shuttling of RNA via exosomes may occur between individuals, during kissing or breastfeeding.

## Competing interests

The authors declare no competing financial interests. JL, KE, AB, MS and HV are co-owners of a patent for the use of exosomes as vectors for gene therapy.

## Authors' contributions

CL designed and carried out the flow cytometry and RNA work for the saliva and breast milk exosomes, conducted the electron microscopy and Western blot experiments for breast milk exosomes and performed the uptake experiments and prepared the manuscript; VSA carried out the flow cytometry and RNA work for plasma exosomes and prepared the manuscript; KE designed the flow cytometry and designed and conducted the electron microscopy for saliva and plasma exosomes; ME and PTP conducted RNA work for breast milk exosomes; AB and MS participated in the planning and designing of the experiment; SG provided knowledge regarding breast milk exosomes; JL conceived of the study and participated in the preparation of the manuscript; HV designed and coordinated experiments and helped prepare sections of the manuscript. All authors read and approved the final manuscript.

## References

[B1] Théry C Zitvogel L Amigorena S Exosomes: composition, biogenesis and function Nat Rev Immunol 2002 2 569 579 12154376 10.1038/nri855

[B2] Théry C Regnault A Garin J Wolfers J Zitvogel L Ricciardi-Castagnoli P Raposo G Amigorena S Molecular Characterization of Dendritic Cell-derived Exosomes: Selective Accumulation of the Heat Shock Protein hsc73 J Cell Biol 1999 147 599 610 10545503 10.1083/jcb.147.3.599 PMC2151184

[B3] Raposo G Nijman HW Stoorvogel W Liejendekker R Harding CV Melief CJM Geuze HJ B Lymphocytes Secrete Antigen-presenting Vesicles J Exp Med 1996 183 1161 1172 10.1084/jem.183.3.1161 8642258 PMC2192324

[B4] Blanchard N Lankar D Faure F Regnault A Dumont C Raposo G Hivroz C TCR Activation of Human T Cells Induces the Production of Exosomes Bearing the TCR/CD3/ζ Complex J Immunol 2002 168 3235 3241 11907077 10.4049/jimmunol.168.7.3235

[B5] Raposo G Tenza D Mecheri S Peronet R Bonnerot C Desaymard C Accumulation of Major Histocompatibility Complex Class II Molecules in Mast Cell Secretory Granules and Their Release upon Degranulation Mol Biol Cell 1997 8 2631 2645 9398681 10.1091/mbc.8.12.2631 PMC25733

[B6] Wolfers J Lozier A Raposo G Regnault A Théry C Masurier C Flament C Pouzieux S Faure F Tursz T Tumor-derived exosomes are a source of shared tumor rejection antigens for CTL cross-priming Nat Med 2001 7 297 303 10.1038/85438 11231627

[B7] Van Niel G Raposo G Candalh C Boussac M Hershberg R Cerf-Bensussan N Heyman M Intestinal Epithelial Cells Secrete Exosome-like Vesicles Gastroenterology 2001 121 337 349 10.1053/gast.2001.26263 11487543

[B8] Caby MP Lankar D Vincendeau-Scherrer C Raposo G Bonnerot C Exosomal-like vesicles are present in human blood plasma Int Immunol 2005 17 879 887 10.1093/intimm/dxh267 15908444

[B9] Pisitkun T Shen R-F Knepper MA Identification and proteomic profiling of exosomes in human urine PNAS 2004 101 13368 13373 10.1073/pnas.0403453101 15326289 PMC516573

[B10] Andre F Schartz NEC Movassagh M Flament C Pautier P Morice P Pomel C Lhomme C Escudier B Le Chevalier T Malignant effusions and immunogenic tumour-derived exosomes Lancet 2002 360 295 305 10.1016/S0140-6736(02)09552-1 12147373

[B11] Skriner K Adolph K Jungblut PR Burmester GR Association of Citrullinated Proteins With Synovial Exosomes Arthritis Rheum 2006 54 3809 3814 10.1002/art.22276 17133577

[B12] Admyre C Johansson SM Qazi KR Filen J-J Lahesmaa R Norman M Neve EPA Scheynius A Gabrielsson S Exosomes with Immune Modulatory Features Are Present in Human Breast Milk J Immunol 2007 179 1969 1978 17641064 10.4049/jimmunol.179.3.1969

[B13] Admyre C Grunewald J Thyberg J Gripenbäck S Tornling G Eklund A Scheynius A Gabrielsson S Exosomes with major histocompatibility complex class II and co-stimulatory molecules are present in human BAL fluid Eur Respir J 2003 22 578 583 10.1183/09031936.03.00041703 14582906

[B14] Gatti J-L Métayer S Belghazi M Dacheux F Dacheux J-L Identification, Proteomic Profiling, and Origin of Ram Epididymal Fluid Exosome-Like Vesicles Biol Reprod 2005 72 1452 1465 10.1095/biolreprod.104.036426 15635128

[B15] Ogawa Y Kanai-Azuma M Akimoto Y Kawakami H Yanoshita R Exosome-Like Vesicles with Dipeptidyl Peptidase IV in Human Saliva Biol Pharm Bull 2008 31 1059 1062 10.1248/bpb.31.1059 18520029

[B16] Chaput N Schartz NEC Andre F Zitvogel L Exosomes for immunotherapy of cancer Adv Exp Med Biol 2003 532 215 221 12908560 10.1007/978-1-4615-0081-0_17

[B17] Morse MA Garst J Osada T Khan S Hobeika A Clay TM Valente N Shreeniwas R Sutton MA Delcayre A A phase I study of dexosome immunotherapy in patients with advanced non-small cell lung cancer J Transl Med 2005 3 9 10.1186/1479-5876-3-9 15723705 PMC551593

[B18] Temchura VV Tenbusch M Nchinda G Nabi G Tippler B Zelenyuk M Wildner O Überla K Kuate S Enhancement of immunostimulatory properties of exosomal vaccines by incorporation of fusion-competent G protein of vesicular stomatitis virus Vaccine 2008 26 3662 3672 10.1016/j.vaccine.2008.04.069 18538453 PMC7115564

[B19] Parolini I Federici C Raggi C Lugini L Palleschi S De Milito A Coscia C Iessi E Logozzi MA Colone M Microenvironmental pH is a key factor for exosome traffic in tumor cells J Biol Chem 2009 284 34211 34222 10.1074/jbc.M109.041152 19801663 PMC2797191

[B20] Valadi H Ekström K Bossios A Sjöstrand M Lee JJ Lötvall JO Exosome-mediated transfer of mRNAs and microRNAs is a novel mechanism of genetic exchange between cells Nat Cell Biol 2007 9 654 659 10.1038/ncb1596 17486113

[B21] Taylor DD Gercel-Taylor C MicroRNA signatures of tumor-derived exosomes as diagnostic biomarkers of ovarian cancer Gynecol Oncol 2008 110 13 21 10.1016/j.ygyno.2008.04.033 18589210

[B22] Michael A Bajracharya SD Yuen PST Zhou H Star RA Illei GG Alevizos I Exosomes from human saliva as a source of microRNA biomarkers Oral Dis 2010 16 34 38 10.1111/j.1601-0825.2009.01604.x 19627513 PMC2844919

[B23] Palanisamy V Sharma S Deshpande A Zhou H Gimzewski J Wong DT Nanostructural and Transcriptomic Analyses of Human Saliva Derived Exosomes PLoS ONE 2010 5 e8577 10.1371/journal.pone.0008577 20052414 PMC2797607

[B24] Morelli AE Larregina AT Shufesky WJ Sullivan MLG Stolz DB Papworth GD Zahorchak AF Logar AJ Wang Z Watkins SC Endocytosis, intracellular sorting, and processing of exosomes by dendritic cells 2004 104 3257 3266 15284116 10.1182/blood-2004-03-0824

[B25] Obregon C Rothen-Rutishauser B Gerber P Gehr P Nicod LP Active Uptake of Dendritic Cell-Derived Exovesicles by Epithelial Cells Induces the Release of Inflammatory Mediators through a TNF-{alpha}-Mediated Pathway 2009 175 696 705 19628765 10.2353/ajpath.2009.080716 PMC2715287

[B26] Lodish HF Zhou B Liu G Chen CZ Micromanagement of the immune system by microRNAs Nat Rev Immunol 2008 8 120 130 10.1038/nri2252 18204468

[B27] García JM García V Peña C Domínguez G Silva J Diaz R Espinosa P Citores MJ Collado M Bonilla F Extracellular plasma RNA from colon cancer patients is confined in a vesicle-like structure and is mRNA-enriched RNA 2008 14 1424 1432 18456845 10.1261/rna.755908 PMC2441977

[B28] Skog J Würdinger T van Rijn S Meijer DH Gainche L Miguel S-E Curry WT Jr Carter BS Krichevsky AM Breakefield XO Glioblastoma microvesicles transport RNA and proteins that promote tumour growth and provide diagnostic biomarkers Nat Cell Biol 2008 10 1470 1476 10.1038/ncb1800 19011622 PMC3423894

[B29] Kramer MS Chalmers B Hodnett ED Sevkovskaya Z Dzikovich I Shapiro S Collet J-P Vanilovich I Mezen I Ducruet T Promotion of Breastfeeding Intervention Trial (PROBIT): A Randomized Trial in the Republic of Belarus 2001 285 413 420 11242425 10.1001/jama.285.4.413

[B30] Kapsogeorgou EK Abu-Helu RF Moutsopoulos HM Manoussakis MN Salivary Gland Epithelial Cell Exosomes: A source of Autoantigenic Ribonucleoproteins Arthritis Rheum 2005 52 1517 1521 10.1002/art.21005 15880835

